# Risk factors of enteral feeding intolerance in severe acute pancreatitis patients: A protocol for systematic review and meta-analysis

**DOI:** 10.1097/MD.0000000000025614

**Published:** 2021-05-07

**Authors:** Hongyun Fan, Chunchun Yang, Zhiying Duan, Xiaohui Huo, Yang Yang

**Affiliations:** a The First Hospital of Hebei Medical University, Shijiazhuang; b Tangshan Hongci Hospital, Tangshan, Hebei Province, China.

**Keywords:** feeding intolerance, meta-analysis, protocol, risk factors, severe acute pancreatitis

## Abstract

**Background:**

Patients with severe acute pancreatitis (SAP) have gastrointestinal dysfunction, and enteral nutrition intolerance is easy to occur during the implementation of enteral nutrition, which leads to the suspension or termination of enteral nutrition. Enteral nutrition cannot tolerate the influence of many factors. At present, there is a lack of analysis on the influencing factors of enteral nutrition intolerance in patients with SAP. Therefore, this study analyzed the factors of enteral nutrition intolerance in patients with SAP by meta-analysis, to provide a basis for the protection of enteral nutrition in patients with SAP.

**Methods:**

Databases (PubMed, Embase, Cochrane Library, Web of Science, China Biology Medicine Database, China National Knowledge Infrastructure, China Science and Technology Journal Database, and Wanfang) were searched using index words to find relevant studies published before March 2021. Meta-analyses of relative risk were performed for the identification of risk factors.

**Results:**

We will disseminate the findings of this systematic review and meta-analysis via publications in peer-reviewed journals.

**Conclusion:**

This study systematically reviewed the existing evidence and determined the incidence and predictors of enteral nutrition intolerance in patients with SAP.

## 1 Introduction

Severe acute pancreatitis (SAP) is a common clinical critical disease with rapid progress and high mortality.^[1,2]^ SAP patients are in a state of high decomposition, which is easy to be associated with gastrointestinal dysfunction, and induce infection and abdominal abscess, resulting in malnutrition.^[3–5]^ Previous studies have shown that parenteral nutrition can provide metabolic needs for the body, reduce the burden on the pancreas, and improve the prognosis.^[6]^ However, with the application of parenteral nutrition, the adverse reactions after treatment are becoming more and more prominent. It was found that with the gradual extension of parenteral nutrition time, the damage of gastrointestinal function and adverse reactions gradually increased, and even induced multiple organ function damage.^[7]^

In recent years, enteral nutrition has been given sufficient attention in the treatment of critically ill patients. Enteral nutrition can not only maintain the homeostasis of intestinal bacteria and keep the intestinal epithelium intact, but also repair the injured intestinal tract and reduce the intestinal injury.^[8,9]^ The clinical practice guidelines of the American Society for Parenteral Nutrition suggest that critically ill patients should receive enteral nutrition as soon as possible.^[10]^ Studies have shown that early enteral nutrition can effectively improve the nutritional status of critically ill patients.^[11–13]^ However, during the period of enteral nutrition, patients often have feeding intolerance (diarrhea, abdominal distension, nausea and vomiting, gastric retention, etc). The incidence of enteral nutrition intolerance is 30.5% to 65.7%.^[14,15]^ This leads to the suspension of enteral nutrition and the inability of patients to take in adequate energy and nutrients, which seriously affects the nutritional status of patients. SAP is a common acute and critical illness in clinic.^[16]^ Due to massive extravasation of pancreatic juice, it can lead to multiple organ function damage, especially gastrointestinal function damage, which can easily lead to enteral nutrition intolerance.^[17,18]^ Therefore, how to avoid the occurrence of feeding intolerance in SAP patients needs to be studied and discussed by nurses.

At present, there are different reports on the influencing factors of enteral nutrition intolerance in patients with SAP.^[19–24]^ The purpose of this study was to analyze the risk factors of enteral nutrition intolerance in patients with SAP by Meta, to provide reference for nurses to prevent and manage enteral nutrition intolerance in patients with SAP.

## 2 Methods

### 2.1 Study registration

This protocol has been registered on Open Science Framework grant number (registration number: DOI 10.17605/OSF.IO/YXJSM). This report will be based on the preferred reporting items for systematic review and meta-analysis protocols.^[25]^

### 2.2 Eligibility criteria

#### 2.2.1 Inclusion criteria

1. Original studies on the effect of enteral nutrition intolerance in patients with SAP;
2. Patients diagnosed with SAP, age ≥ 18 years old, regardless of gender and race;
3. Studies whose results involve the specific values of relative risk (RR) and 95% confidence interval (95% CI) of risk factors.

#### 2.2.2 Exclusion criteria

1. Studies that only have the abstract and cannot carry on the research of data extraction;
2. Studies published repeatedly;
3. Review, systematic review, conference, animal experiments, and other literatures;
4. Studies that are unable to carry out the research of data conversion.

### 2.3 Search strategy

Studies delineating the enteral feeding intolerance in SAP patients were consistently and exhaustively searched using medical specialty databases of PubMed, Embase, Cochrane Library, Web of Science, China Biology Medicine Database, China National Knowledge Infrastructure, China Science and Technology Journal Database, and Wanfang. The retrieval time was from the establishment of the database to March 2021. These search terms are summarized in Table 1.

**Table 1:** Search strategy used in PubMed database.

Number	Search terms
#1	Pancreatitis[MeSH]
#2	Pancreatitides[Title/Abstract]
#3	or/1–2
#4	Enteral Nutrition[MeSH]
#5	Enteral Feeding[Title/Abstract]
#6	Force Feeding[Title/Abstract]
#7	Nutrition, Enteral[Title/Abstract]
#8	Tube Feeding[Title/Abstract]
#9	Gastric Feeding Tubes[Title/Abstract]
#10	Feeding Tube, Gastric[Title/Abstract]
#11	Feeding Tubes, Gastric[Title/Abstract]
#12	Feeding, Enteral[Title/Abstract]
#13	Feeding, Force[Title/Abstract]
#14	Feeding, Tube[Title/Abstract]
#15	Feedings, Forcee[Title/Abstract]
#16	Force Feedings[Title/Abstract]
#17	Gastric Feeding Tube[Title/Abstract]
#18	Tube, Gastric Feeding[Title/Abstract]
#19	Tubes, Gastric Feeding[Title/Abstract]
#20	or/4–19
#21	Intolerance[Title/Abstract]
#22	Risk factor[Title/Abstract]
#23	Risk assessment[Title/Abstract]
#24	Multivariate analysis[Title/Abstract]
#25	Multivariable logistic regression[Title/Abstract]
#26	or/22–25
#27	#3 and #20 and #21 and #26

### 2.4 Study selection

**Figure 1:**
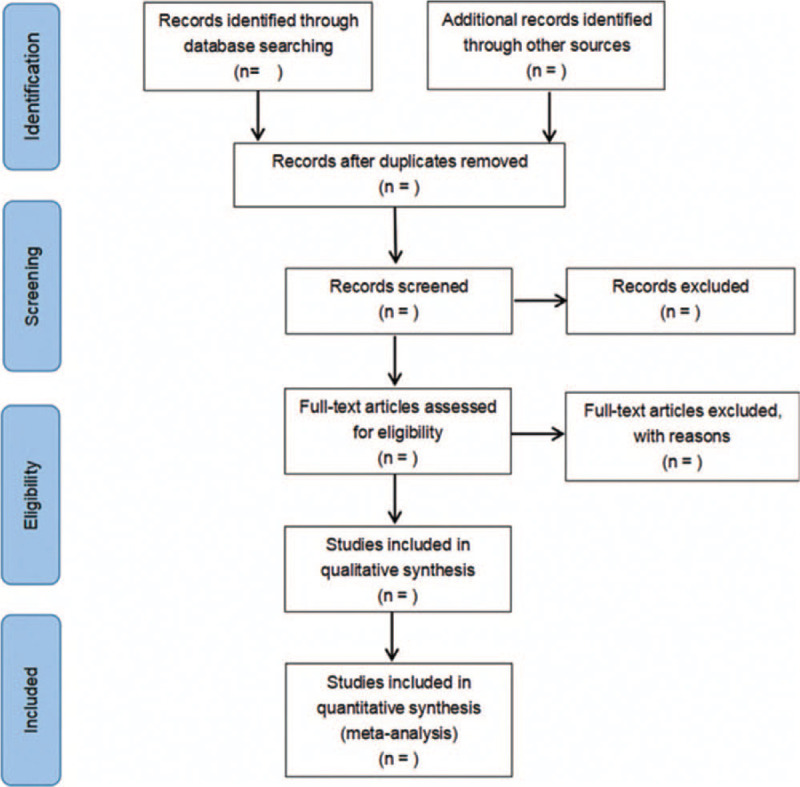
Flow chart of study selection.

The process of the selection is exhibited in Figure 1. First, the retrieved literature is de-duplicated by EndNote. Second, 2 evaluators independently read the remaining literature titles and abstracts for preliminary screening. According to the inclusion and exclusion criteria of the literature, the full text of the literature after preliminary screening was rescreened. It is up to the third party to decide whether to include the controversial literature in the process of literature screening.

### 2.5 Data extraction

The data of the included literature were extracted independently by 2 evaluators. The specific contents of data extraction include: author, year of publication, type of study, sex, age, total sample size, number of patients in case group and control group, incidence of feeding intolerance, and risk factors of feeding intolerance.

### 2.6 Assessment of the risk of bias

Methodological quality of all studies was assessed using the Newcastle-Ottawa scale (NOS).^[26]^ NOS score ≥ 6 means that the literature quality is better.

### 2.7 Data analysis

We will use RR and 95% CI to represent. If there are no findings of statistical heterogeneity, the Mantel-Haenszel fixed effect model is adopted for data synthesis.^[27]^ If there is significant statistical heterogeneity, we will apply the DerSimonian-Laird random effect model.^[28]^ The data analysis of this study will be conducted through Review Manager 5.3 software.

### 2.8 Assessment of heterogeneity

The magnitude of heterogeneity in the results was determined by χ2 test and *I*^*2*^ quantitative analysis. When *P* < .1, and (or) *I*^*2*^ > 50%, the random effect model is adopted for the combined analysis. Otherwise, the fixed effect model is adopted.

### 2.9 Subgroup analysis

According to the study type, age, and region, we make a subgroup analysis.

### 2.10 Sensitivity analysis

Sensitivity analysis is to test the stability of meta results by changing the data analysis model.

### 2.11 Assessment of reporting biases

Publication bias is analyzed by funnel chart.

### 2.12 Management of missing data

The research was on the defects of the original data. We contacted the author by email and asked for the original data. If the original data were not available, we would analyze the existing data.

### 2.13 Ethical review and informed consent of patients

The content of this article does not involve moral approval or ethical review and will be presented in print or at relevant conferences.

## 3 Discussion

SAP is a common acute abdomen in the Department of Gastroenterology. It occurs rapidly and is critically ill, with a fatality rate as high as 20%.^[29]^ During SAP, the body is in a state of stress and high catabolism, and the energy consumption increases sharply, which is up to 1.2 to 1.5 times of the basic value and is prone to malnutrition and decreased immunity.^[30]^ Timely and efficient nutritional support has become an important link in the treatment of SAP. Enteral nutrition, as a nutritional support method in line with normal physiology, can not only avoid the stimulation of the pancreas by nutrient solution, but also achieve the purpose of pancreatic rest. It also helps to maintain intestinal mucosal epithelial barrier function, reduce intestinal bacterial translocation and endotoxin release, reduce inflammatory response, and improve patient prognosis.^[31]^

Patients with SAP have gastrointestinal dysfunction and are prone to enteral nutrition intolerance, which leads to the suspension or termination of enteral nutrition. How to individually predict, evaluate, and prevent the occurrence of enteral nutrition intolerance in patients with SAP is becoming an urgent problem to be solved in clinical work. In this study, meta-analysis was used to analyze the risk factors of enteral nutrition intolerance in patients with SAP, so as to provide evidence for clinical individualized intervention and risk prediction.

## Author contributions

**Conceptualization:** Hongyun Fan and Zhiying Duan.

**Data collection:** Hongyun Fan and Chunchun Yang.

**Funding acquisition:** Yang Yang.

**Resources:** Chunchun Yang and Zhiying Duan.

**Software:** Xiaohui Huo.

**Supervision:** Xiaohui Huo and Yang Yang.

**Writing – original draft:** Hongyun Fan and Chunchun Yang.

**Writing – review & editing:** Hongyun Fan and Yang Yang.
